# Rivastigmine improves dual-task gait velocity in patients with Alzheimer’s disease

**DOI:** 10.1186/s12883-021-02098-8

**Published:** 2021-02-10

**Authors:** Hideki Shimura, Aiba Saiko, Akito Hayashi, Nobutaka Hattori, Takao Urabe

**Affiliations:** 1 Department of Neurology, Juntendo University Urayasu Hospital, 2-1-1 Tomioka, Urayasu, Chiba, 279-0021 Japan; 2 Department of Rehabilitation, Juntendo University Urayasu Hospital, Chiba, Japan; 3 Department of Neurology, Juntendo University School of Medicine, Tokyo, Japan

## Abstract

**Background:**

Gait impairments are common in patients with Alzheimer’s disease. Cholinesterase inhibitors are used to treat the symptoms of patients with Alzheimer’s disease, but they have not been shown to reduce the severity of Alzheimer’s disease-related gait disorders.

**Methods:**

This was a prospective, single-arm, open-label, non-randomized study. The aim of the present study was to determine the effect of the acetylcholinesterase inhibitor rivastigmine on gait in 21 newly diagnosed patients with mild to moderate Alzheimer’s disease. The outcome variables were velocity, stride length, and cadence during single-task and dual-task gait trials. The subjects were also assessed with the Mini-Mental State Examination, Alzheimer’s Disease Cooperative Study Activities of Daily Living, Functional Assessment Staging, and Geriatric Depression Scale.

**Results:**

After 12 weeks of treatment with rivastigmine, gait velocity was significantly improved in the dual-task gait trials; gait velocity was increased from 40.59 ± 13.59 m/min at baseline to 46.88 ± 12.73 m/min when counting backward from 100 in steps of 7 while walking, and gait velocity was increased from 37.06 ± 15.57 m/min at baseline to 42.03 ± 14.02 m/min when naming animals while walking. In the single-task gait trials, which consisted only of walking at their usual pace or at a fast pace, gait velocity was not increased by rivastigmine administration.

**Conclusion:**

Our findings indicated that rivastigmine improved gait in subjects with mild to moderate Alzheimer’s disease during dual-task trials. The observed enhancement of dual-task gait might be caused by an improvement of cognitive function rather than motor function.

**Trial registration:**

UMIN, UMIN000025869. Registered December 16, 2016, https://upload.umin.ac.jp/cgi-open-bin/icdr/ctr_view.cgi?recptno=R000029744

## Background

Alzheimer’s disease (AD) is the most common neurodegenerative disorder and one of the leading causes of death in old age [1]. AD affects a variety of functional areas, including cognitive and motor functions [1]. Recently, the relationship between motor activity and dementia has received increasing research attention [2]. Gait abnormalities are commonly observed in patients with AD and increase in frequency and severity over time [3]. Gait disorders decrease mobility and increase the risk of falling [4]. The consequences of gait disorders and associated falls can be severe, including fractures, worsening of mobility, loss of independence, and increased cardiovascular morbidity and mortality [5]. The presence of gait abnormalities in AD is important for predicting faster cognitive decline, institutionalization, and death [6].

Gait disturbance in patients with AD is particularly evident under dual-task conditions [7, 8], e.g., in simple tasks performed while walking such as counting backward or in more complex tasks such as verbal fluency [2]. As dual-task gait assessments isolate the cognitive cost of maintaining a safe gait while distracted, they have been used to indicate that impairments in cognition lead to deficits in gait control that are independent of the decline in muscle strength and osteoarticular function that accompanies aging [9]. Specifically, higher-level motor control requires cognition to produce the complex motor responses that are adapted to multiple sensory inputs and environmental challenges. Thus, impaired cognitive abilities, especially attention and executive function, compromise postural and gait stability [10]. The dual-task paradigm can be used to study the allocation of attentional resources during a motor task and to separate the cognitive and motor components of executing a movement [2].

Previous studies suggest that cholinesterase inhibitors (ChEIs), a current treatment for the symptoms of AD, may improve gait performance [11–13]. Since, cholinesterase inhibition improves attention and executive function in patients with AD [14], which are both associated with gait quality [15], we hypothesized that the acetylcholinesterase inhibitor (AChEI) rivastigmine would improve gait quality, as quantified by gait velocity, stride length, and cadence, in single- and dual-task gait trials.

## Methods

### Participant flow

**Fig. 1:**
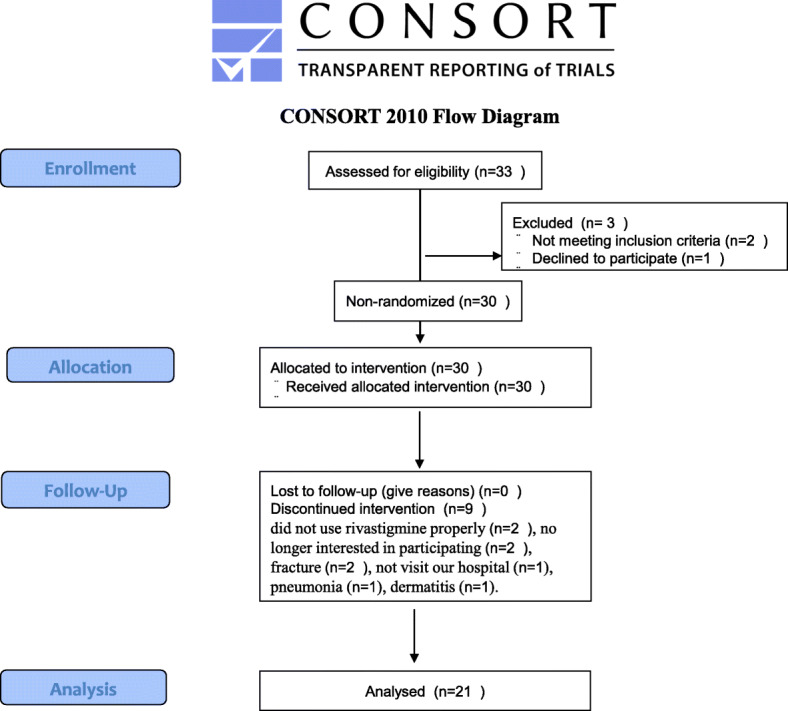
CONSORT 2010 Flow Diagram

Patient flow in the study is described in Fig. 1.

### Participants and procedure

The participants were recruited from December 2016 to June 2018 from the Memory Clinic at Juntendo University Urayasu Hospital. Newly diagnosed older adults with mild to moderate AD who were prescribed a rivastigmine patch were approached for recruitment. The participants were eligible to enter the trial if they met all of the following criteria at baseline: diagnosis of probable AD according to the criteria of the National Institute of Neurologic and Communicative Disorder and Stroke-AD and Related Disorders Association; Mini-Mental State Examination (MMSE) score greater than 14 to be considered mild or moderate AD; had a caregiver who could assist the participant with medication; and had the ability to walk independently, i.e., without a walking aid or without assistance from other people. Participants aged 65 years or older were recruited. Subjects were not included if they had a history of head trauma with loss of consciousness and concomitant medication including benzodiazepines or antipsychotics. We also excluded patients with any neurological disorder with motor residual deficits including parkinsonism, stroke, and polyneuropathy that influenced their ability to carry out a walking task. All participants received magnetic resonance imaging or computed tomography, and an N-isopropyl-p-(123 I)-iodoamphetamine single photon emission computed tomography cerebral blood flow test was performed in all participants. The absence of a large lesion, i.e., infarction, tumor, and inflammation, was confirmed in all participants. We also confirmed the presence of hypoperfusion in the parietal lobe, which is consistent with a diagnosis of AD.

### Design

Baseline cognitive and gait assessments were performed on the day before rivastigmine was administered. The participants received 9 mg/day rivastigmine for 4 weeks and 18 mg/day by transdermal patch for the subsequent 8 weeks of follow-up. Gait, cognitive, and psychological functions and AChE activity in plasma were analyzed at baseline, 4 weeks, and 12 weeks.

### Cognitive and psychological assessments

We administered the MMSE, Alzheimer’s Disease Cooperative Study Activities of Daily Living (ADCS-ADL), Functional Assessment Staging (FAST), and Geriatric Depression Scale (GDS) to all participants.

### Gait assessment

Quantitative gait variables were assessed using a portable gait rhythmograph (MG-M 1110; LSI Medience Corporation, Tokyo, Japan), which is a small device (8 × 6 × 2 cm; weight, 80 g) with an accelerometer. The portable gait rhythmograph measures the acceleration accompanied by limb and trunk movements and acceleration induced by step-in and kick-off during gait in three dimensions (*ax*, *ay*, *az*). Its accuracy has been verified previously [16].

Gait speed, average step length, acceleration, and cadence, which is the number of steps per minute, were measured in single- and dual-task gait trials. The single-task trials consisted of normal gait at a self-selected usual pace and fast gait. For the dual-task trials, the participants walked while counting backward from 100 in steps of 7 or while naming animals aloud. Ten-meter-walk tests were performed on the level floor of the hospital in the presence of two investigators (SA and HS). The order of gait analysis was normal gait for the first trial, fast gait for the second trial, counting backward by seven for the third trial, and animal naming for the fourth trial. The participants briefly practiced dual-task walking before the first assessment. The participants did not practice dual-task walking during the study period.

### Gait analysis

Using the portable gait rhythmograph, gait-induced acceleration was extracted from limb and trunk movements with an automatic gait detection algorithm [16, 17]. The portable gait rhythmograph performs three-dimensional measurements of acceleration associated with voluntary limb and trunk movements, heel strike, and toe-off when walking. Data were collected at a sampling frequency of 100 Hz and stored on a microSD card in the device for subsequent analysis. When recording was complete, the absolute values of the acceleration vectors were calculated and displayed graphically on a PC.

### Statistical analysis

A paired Student’s t-test was used to detect group differences before and at 12 weeks after rivastigmine treatment for gait speed, average step length, acceleration, and cadence in single- and dual-task gait trials. Statistical significance was considered for *P*-values less than 0.05.

### Standard protocol approval registration and patient consent

This study was conducted in accordance with the ethical standards set forth in the Declaration of Helsinki (1983). This study was approved by the local ethics committee of Juntendo University Urayasu Hospital. Each participant provided written informed consent. If a participant had impaired decisional capacity, their family provided consent and the participants provided assent.

## Results

### Subject characteristics and follow-up

Thirty participants with mild to moderate AD were initially enrolled, of which 21 completed the full study and were included in the analyses. The nine subjects who withdrew did so for the following reasons: two did not use rivastigmine properly, two were no longer interested in participating, two suffered a fracture, one did not visit our hospital because their caregiver suffered a fracture, one developed mild pneumonia, and one could not continue using the rivastigmine patch because of dermatitis.

### Adverse events

One participant collided with another person while riding a crowded train and fractured his left humerus; he underwent open fusion for the fracture and recovered without sequelae. Another patient suffered a lumbar compression fracture by bumping into another person while walking; she was hospitalized for 1 month and recovered without sequelae. Both of these patients continued to use rivastigmine. One participant was withdrawn due to medication intolerance by dermatitis (as stated in the previous section).

### Baseline characteristics and outcomes after intervention

The baseline characteristics of the subjects are presented in Table 1. At baseline, the subjects had a mean single-task gait velocity of 59.21 m/min, which is considered normal gait velocity. Dual-task gait velocity while counting backward by seven was decreased to 40.59 m/min compared to single-task gait, and decreased to 37.06 m/min when naming animals (Table 1).

**Table 1:** Baseline Characteristics of the Subjects

Age (mean, SD)		79.03 (6.89)	
Sex (female; n, %)		13 (61.9%)	
Mini-Mental State Examination (mean, SD)		19.62 (4.71)	
Geriatric Depression Scale (mean, SD)		2.76 (1.09)	
Functional Assessment Staging (mean, SD)		4.24 (0.83)	
Alzheimer’s Disease Cooperative Study Activities of Daily Living (mean, SD)		63.14 (8.6)	
Single-task gait trials	velocity (m/min)	stride length (cm)	cadence (steps/min)
Normal gait (mean, SD)	59.21 (12.21)	50.11 (7.52)	117.69 (12.26)
Fast gait (mean, SD)	70.33 (16.06)	54.64 (8.58)	127.69 (13.37)
Dual-task gait trials			
Counting backward by 7	40.59 (13.59)	46 (9.29)	95.38 (23.88)
Naming animals	37.06 (15.57)	46.25 (9.24)	80.46 (27.15)

*SD* Standard deviation

### Gait assessment

In single-task gait analysis, the gait velocity, stride length, and cadence of the subjects did not significantly change from baseline to after 12 weeks intervention. In dual-task gait analysis, the subjects increased their gait velocity when counting backward by seven from 40.59 ± 13.59 at baseline to 46.88 ± 12.73 m/min at 12 weeks (*p* = 0.025), and their gait velocity when naming animals increased from 37.06 ± 15.57 at baseline to 42.03 ± 14.02 m/min at 12 weeks (*p* = 0.036). The subjects also increased their cadence when counting backward by seven from 89.37 ± 24.64 at baseline to 99.35 ± 18.02 at 12 weeks (*p* = 0.048). Stride length did not significantly change from baseline to after 12 weeks intervention (Table 2).

**Table 2:** Gait Assessment

	Baseline	12 weeks	*p*-value (12 weeks vs. baseline)
Rivastigmine	0 mg	18 mg/day	
Single-task gait trials			
Normal gait (mean, SD)			
velocity (m/min)	59.21 (12.21)	59.82 (11.46)	0.733
stride length (cm)	50.11 (7.52)	51.68 (7.27)	0.111
cadence (steps/min)	117.69 (12.26)	115.55 (13.26)	0.412
Fast gait (mean, SD)			
velocity (m/min)	70.33 (16.06)	69.44 (12.62)	0.691
stride length (cm)	54.64 (8.58)	55.5 (7.95)	0.367
cadence (steps/min)	127.69 (13.37)	125.11 (13.17)	0.406
Dual-task gait trials			
Counting backward by 7 s			
velocity (m/min)	40.59 (13.59)	46.88 (12.73)	0.025*
stride length (cm)	46 (9.29)	46.37 (6.61)	0.716
cadence (steps/min)	89.37 (24.64)	99.35 (18.02)	0.048*
Naming animals			
velocity (m/min)	37.06 (15.57)	42.03 (14.02)	0.036*
stride length (cm)	46.25 (9.24)	47.55 (7.46)	0.199
cadence (steps/min)	80.46 (27.15)	88.76 (23.42)	0.076

Paired sample t-test: *p < 0.05

*SD* Standard deviation

### Cognitive and psychological assessments

The subjects showed an improvement in the MMSE score from 19.62 ± 4.71 at baseline to 20.29 ± 4.66 at 12 weeks (*p* = 0.001). However, there was no significant improvement of the scores for FAST, GDS, and ADCS-ADL (Table 3).

**Table 3:** Cognitive and Psychological Assessments and Acetylcholinesterase Activity

	Baseline	12 weeks	*p*-value (12 weeks vs. baseline)
MMSE	19.62 ± 4.71	20.29 ± 4.66	0.001*
FAST	4.24 ± 0.83	4.14 ± 0.85	0.16
ADCS-ADL	63.14 ± 8.6	63.29 ± 8.17	0.48
GDS	2.76 ± 1.09	2.86 ± 1.11	0.16
AChE activity (IU/L)	276.81 ± 56.28	159.81 ± 60.25	< 0.001*

Paired sample t-test: *p < 0.05

*AChE* Acetylcholinesterase, *ADCS-ADL* Alzheimer’s Disease Cooperative Study Activities of Daily Living, *FAST* Functional Assessment Staging, *GDS* Geriatric Depression Scale, *MMSE* Mini-Mental State Examination

### AChE activity

AChE activity was significantly decreased from 276.81 ± 56.28 IU/L at baseline to 159.81 ± 60.25 IU/L at 12 weeks (Table 3), indicating that the subjects took rivastigmine as prescribed.

## Discussion

The present study showed that rivastigmine improved gait velocity under dual-task conditions in subjects with mild to moderate AD. Conversely, rivastigmine did not improve gait velocity, cadence, and stride length under single-task conditions.

AChEIs stabilize cognitive function and delay functional decrease [18]. Although it is not clear by which mechanism AChEIs have this effect, it is recognized that they improve not only cognitive function but also motor function. Acetylcholine has an important role in cognitive function and in controlling gait and balance [19]. AChEIs are thought to contribute to the initiation and maintenance of gait by improving executive function and attention and the control of step length and gait velocity. A limited number of studies have shown that ChEIs improved gait performance in patients with AD. Donepezil significantly improved gait velocity in subjects with mild AD under single- and dual-task conditions measured using an electronic walkway [20]. Galantamine improved dual-task stride time in a small number of patients with moderate AD [12]. In the present study, rivastigmine significantly improved gait velocity under dual-task conditions in 21 subjects with mild to moderate AD.

Cognitive enhancers could improve gait by a number of mechanisms. Cognitive function and neural control of gait share brain cortical networks and neurotransmitters [2]. The neurotransmitter acetylcholine has an important role in cognitive function and in controlling gait and balance [13]. Specifically, thalamic activity is derived mainly from the brainstem pedunculopontine nucleus, which plays a central role in the generation of movement, gait, and balance control [21]. Cholinergic forebrain projections from the nucleus basalis of Meynert also have a specific role in the control of selective attention, which is an important factor in the cost of dual-tasking while walking in subjects with AD. ChEIs stabilize and improve attention and executive function in patients with AD and other neurodegenerative disorders [22]. There may be cognitive- and non-cognitive-related enhancement mechanisms by which ChEIs improve gait and potentially improve gait performance.

In our study, rivastigmine increased gait velocity in the dual-task trials, but not in the single-task trials. The principle dual-task paradigm involving gait is the creation of an attention-demanding task [23]. A decline in gait performance while performing a dual task when compared to a single task is usually interpreted as interference due to competing demands for attention between both tasks [23]. Thus, gait performance while dual tasking appears to be more dependent on cortical cholinergic levels than while performing a single task [24], suggesting that cognitive enhancement may have affected the increase of gait velocity in our subjects rather than non-cognitive enhancement. The increase in gait velocity during the dual task was mainly due to an increase in cadence, but not stride length, in our study. We speculate that motor function may be more involved in stride length than cognitive function as compared with cadence. Although both the animal naming test and the counting backward by seven test are associated with prefrontal and anterior cortex regions, there was a significant improvement in cadence when counting backward rather than when naming animals. The participants were faster and more accurate when counting backward than when naming animals. Baseline cadence data were better when the participants counted backward than when naming animals, which may be related to the fact that cadence was significantly improved when the participants counted backward. Cadence did improve slightly during the animal naming test, but not significantly so. The reason for this discrepancy between the two dual tasks should be examined in future studies.

Rivastigmine also significantly improved the MMSE score, but this improvement was negligible. Rivastigmine did not significantly improve the scores for FAST, GDS, and ADCS-ADL. The AChE activity of all subjects was decreased. These results indicate that rivastigmine did not greatly improve the cognitive function of the participants in this study.

Some limitations of this study need to be considered. Firstly, we used a single-arm open-label design with no randomization and no placebo group. Future studies will be needed with a placebo group. Second, there is a possibility of a learning effect due to the repetition of the dual tasks and MMSE, which may have affected the results of these tests. Third, the small number of participants from one clinic may be unrepresentative of the general population of patients with AD. Fourth, although we were able to control for changes of gait and treatment, residual confounders (e.g., physical condition or motivation) might still be present.

Two participants suffered a fracture during the study period. The contribution of rivastigmine to fracture cannot be ruled out completely, but we believe that any direct effect is small as both fractures occurred by accidental collision.

## Conclusions

In conclusion, we found a rivastigmine-related increase in gait velocity of patients with AD in dual-task trials. This improvement may contribute to gait function in patients with AD. This study had a single arm design with a small number of participants and the statistical strength of the study was not strong. Double blind randomized placebo-controlled parallel trial testing is necessary to confirm the effectiveness of rivastigmine on gait disorders in patients with AD in the future.

## Data Availability

Not applicable.

## Abbreviations

AChEI: Acetylcholinesterase inhibitor
AD: Alzheimer’s disease
ADCS-ADL: Alzheimer’s Disease Cooperative Study Activities of Daily Living
FAST: Functional Assessment Staging
GDS: Geriatric Depression Scale
MMSE: Mini-Mental State Examination
